# A Frequency-Correcting Method for a Vortex Flow Sensor Signal Based on a Central Tendency

**DOI:** 10.3390/s20185379

**Published:** 2020-09-20

**Authors:** Bin Li, Chengyi Wang, Jie Chen

**Affiliations:** School of Mechatronic Engineering and Automation, Shanghai University, Shanghai 200072, China; sulibin@shu.edu.cn (B.L.); ihere21@shu.edu.cn (C.W.)

**Keywords:** vortex flow sensor, signal processing, central tendency

## Abstract

A vortex flow meter employs a sensor based on the piezoelectric vibration principle to realize vortex signal acquisition, and therefore the measurement results are susceptible to vibration noise. In this paper, the generalized mode method is proposed based on the central tendency characteristic of the vortex signal and combined with the existing filter bank method. The method combining filter bank with the generalized mode is designed and applied in the signal-processing system of the vortex flow meter, which makes up for the defect that the filter bank method cannot filter out the noise in the sub-band. The simulation experiments verify the feasibility and anti-interference performance of the algorithm. Meanwhile, a comparison with two FFT (Fast Fourier Transform) spectrum analysis methods shows that the algorithm designed in this paper requires a smaller sample size and achieves better real-time performance. The actual anti-vibration experiment and calibration experiment verify that the signal-processing system of a vortex flow meter ensures good accuracy and repeatability.

## 1. Introduction

**Figure 1:**
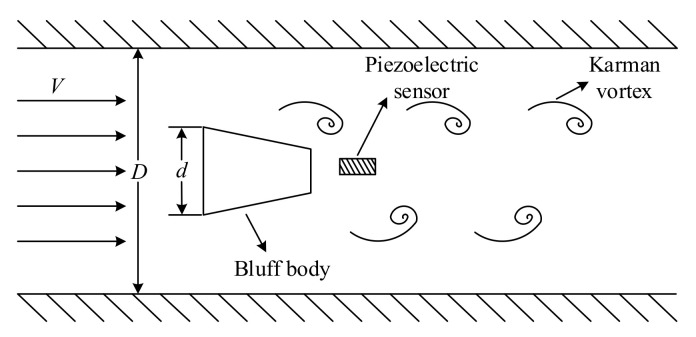
The principle of a vortex flow meter.

Flow measurement is one of the most important components of measurement science and technology. It is closely related to a national economy, national defense construction, and scientific research. Flow measurement is widely used in various fields related to production and lives, including petroleum industry production, chemical agent production, electric power industry production, and so on. With the improvement of production and life quality, the demand for flow measurement accuracy has also been increasing. A vortex flow meter is one of the most widely used flow measurement instruments. Research into the signal-processing method of a vortex flow meter has practical application value and can bring some social and economic benefits. A vortex flow meter has the advantages of wide applicable range, broad measurement range, little pressure loss, and high measurement precision [1,2,3]. Figure 1 shows the principle of a vortex flow meter [4]: a non-streamlined vortex generator is placed in the measured medium, and two rows of staggered vortices known as Kalman vortex and a lift force will be generated when the fluid flows through the generator. The lift force detected by the piezoelectric sensor will be converted into an electric signal.

Research shows that the vortex signal has the following two characteristics:

1. Ideally, the output signal of vortex flow sensor is a sinusoidal wave [5,6] and its frequency is proportional to the flow velocity [7], as shown in Equation (1). Therefore, the flow velocity can be measured by measuring the signal frequency;
  (1) $$f_{v}\;=\left(S_{t}v\right)/d$$
  where $f_{v}\;(\mathrm{Hz}$) is the signal frequency, $\;S_{t}$ is the Strouhal number,$\;v\;\left(\frac{m}{s}\right)$ is the fluid velocity, and $d\;\left(m\right)$ is the width of the bluff body.
2. The vortex signal also satisfies the characteristic that the amplitude is proportional to the frequency square [7], as shown in Equation (2), which is called the amplitude square dependence.
  (2) $$A\propto \rho f_{v}{}^{2}$$
  where $A\left(V\right)$ is the amplitude; $\rho \;(\mathrm{kg}/m^{3})$ is the fluid density.

According to the characteristics mentioned above, it can be seen that both frequency and amplitude of a vortex signal are determined at a specific velocity. This means that a vortex signal has a good central tendency in the frequency domain and there is a prominent peak at the frequency point of the vortex signal in the spectrum.

As vortex flow meter measurement is based on the vibration detection principle, its measurement results are easily interfered with by vibration noise. Moreover, the noise in particular is close to the frequency of vortex signal, making it hard filtered out. The common noises in vortex signal include: 50 Hz power frequency noise, low-frequency noise of 1/5–1/10 vortex frequency, fluid flow noise, periodic vibration noise, and transient impact vibration noise [8]. These noises may exist in the whole frequency band of a vortex signal. Therefore, how to filter out the noise and improve the measurement accuracy has become an essential problem in the research of vortex flow meters.

To solve the difficulties in vortex signal processing, scholars have undertaken extensive explorations. This research is mainly divided into two categories. One is to use classical or new signal-processing methods in other fields for reference and apply them to vortex flow meter system. The other is to design a signal-processing method based on the characteristics of vortex signal.

Methods of the first category mainly include the autocorrelation method, cross-correlation method, empirical mode decomposition method, and wavelet analysis method.

Xu used the autocorrelation function to represent the frequency bandwidth of vortex signal and noise. He identified vortex signal and noise according to their different frequency bandwidth. The results of the liquid flow calibration experiment and mechanical vibration experiment showed that this method performs well [9]. Schlatter established different templates for the vortex signal and noise, and eliminated intense noise through cross-correlation calculation of signal template and noise template. The study improved the measurement accuracy of vortex frequency [10]. Venugopal proposed a double-sensor cross-correlation technique to expand the measurement range of a vortex flow meter. Under the condition of high Reynolds number, the output signal of a single piezoelectric sensor was used to calculate the vortex frequency. Under the condition of low Reynolds number, the output signals of two piezoelectric sensors were used to calculate the fluid velocity through cross-correlation [11]; He also proposed a method based on an EMD (empirical mode decomposition) algorithm to solve the problem that the piezoelectric sensors are greatly affected by vibration interference during small flow measurement. This algorithm has strong robustness and can work stably even under low Reynolds number [12]. Zhang proposed an improved time-frequency analysis method based on EMD and the Hilbert transform to extract a weak vortex signal. EMD was used to filter noise, and the Hilbert transform was used to obtain instantaneous frequency distribution. The vortex frequency was calculated according to the last residual component. This method extended the lower limit of flow measurement of a vortex flow meter [3]. He also used wavelet analysis method to extract the signal from noise, and selected db4 and db5 as the waves of the wavelet transform to filter noise. This method extended the lower limit of flow [2]. Du proposed an algorithm combining wavelet analysis algorithm and single branch reconstruction algorithm for vortex signal processing. The results showed that this algorithm can extract vortex signal from noise at a low flow rate [13].

Although the above methods can achieve good measurement accuracy, the advantages of low power consumption and real-time performance are not obvious due to complex algorithms and large computational load.

Methods of the second category mainly include the FFT (Fast Fourier Transform) spectrum analysis method and the filter bank method.

According to the first characteristic of vortex signal, scholars have proposed various FFT spectrum analysis methods to measure the flow by extracting the frequency of vortex signal. Amadi-Echendu studied the influence of the noise in the working environment on the vortex signal frequency and used the FFT spectrum analysis to calculate the frequency, which improved the measurement accuracy of the flow meter [14]. Zhang proposed a ‘double-window’ algorithm based on quadratic FFT spectrum analysis to solve the problem of fluid disturbed by noise at a low flow rate [15]. Li evaluated the influence of non-integral period sampling and frequency resolution on the vortex signal, and the experiment results showed that the FFT spectrum analysis could improve the measurement capability of fluid at low flow rate [16]. Chen used sparse Fourier transform to analyze the amplitude and frequency of the signal, and the computational complexity of this method is only 1/10 of that of traditional FFT spectrum analysis method [17]. Xu proposed a frequency variance algorithm based on discrete spectrum analysis to identify the vortex signal by judging the variance of signal frequency, improving the anti-vibration performance of the vortex flow meter [18]; he also used FFT spectrum analysis and digital filter to process vortex signal, and combined the autocorrelation function to identify signal and noise. The results of a flow calibration experiment and mechanical vibration experiment showed the good performance of this method [19]; recently, he estimated the power spectrum of vortex signal with FFT spectrum analysis and corrected the peak frequency of the power spectrum with a weighted mean method, which improved the reliability and anti-interference of the signal-processing method [20]. This method represents the latest development of FFT spectrum analysis. However, the measurement results of FFT spectrum analysis are easily affected by the fence effect and spectrum leakage [21,22]. Moreover, there is always a contradiction between the sample size and the frequency resolution, which will affect the real-time performance of signal processing in engineering.

**Figure 2:**
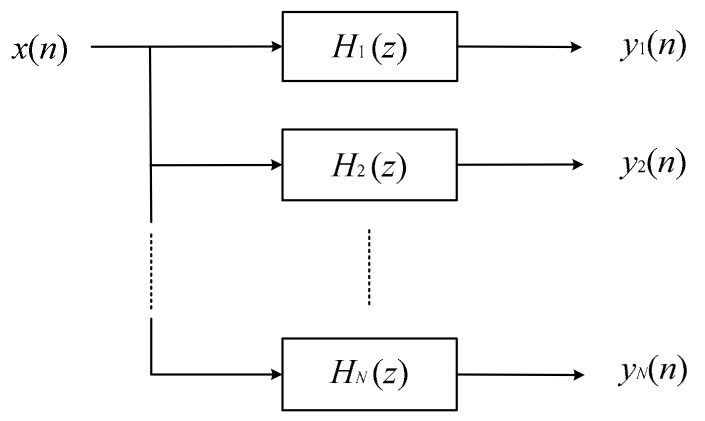
The principle of the filter bank method.

**Figure 3:**
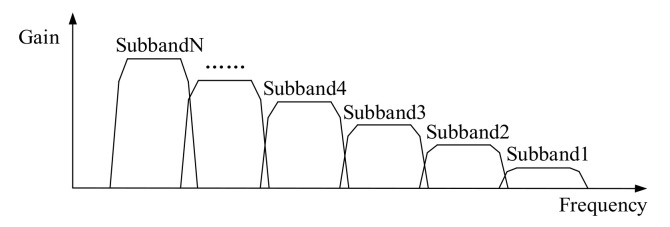
The amplitude-frequency characteristic of the filter bank.

According to the second characteristic of a vortex signal, the products of a vortex flow meter generally adopt the filter bank method to filter the noise by dividing the frequency band of signal processing [23,24]. The basic principle of the filter bank method is shown in Figure 2. A series of parallel filters divide the whole frequency band into N sub-bands, and each sub-band corresponds to a filter, $\;H_{1}\left(z\right),H_{2}\left(z\right),\ldots ,H_{N}\left(z\right)$. After $x\left(n\right)$ passing through the N filters, each sub-band signal can be obtained as $y_{1}\left(n\right),y_{2}\left(n\right),\ldots ,y_{N}\left(n\right)$. The amplitude-frequency characteristic of the filter bank combined with the $1/f^{2}\;$ amplitude squared dependence is shown in Figure 3. After $x\left(n\right)$ passing through the filter bank, the amplitude of the vortex signal fluctuates around a constant at different frequencies. The sinusoidal wave of each sub-band will be transformed into a square wave by pulse shaping. Generally, the cycle data of the output signal of the current channel is averaged by the microprocessor, and its reciprocal is taken as the vortex signal frequency. According to the principle of matching the frequency calculation results with the filtering frequency band, the best filtering channel is selected.

Novel vortex signal processing methods and vortex flow meter products based on the filter bank method have been developed. Fan proposed a novel algorithm of tracking bandpass digital filter with amplitude $1/f^{2}$ attenuation for vortex signal extraction. The system contained a special bandpass filter bank for different diameters and fluid types, and it can adjust the cut-off frequency of the filter bank according to the change of vortex frequency [4]. Rosemount used the amplitude-frequency characteristics of the vortex signal to construct a dedicated bandpass filter. The filter was composed of a low-pass filter and some high-pass filters with different cut-off frequencies. The signal amplitude was attenuated by the low-pass filter with amplitude square dependence so that the amplitude of the filtered vortex signal was maintained at a constant. According to the characteristics of a current vortex signal, the parameters of the filter are optimized and adjusted by the microprocessor [24]. The Yokogawa company used a filter bank to process vortex signal and completed the overall design and implementation of the digital vortex flow meter system. The system used the sub-band filter bank with amplitude square dependence to filter noise. By comparing and distinguishing the amplitude measurement results of the filtered signal, the appropriate filter was selected to complete the final filtering [25]. Shanghai University proposed a filter bank method based on the different characteristics of a vortex signal in low velocity and high velocity. There were only four filter banks, and each filter bank included a low-pass filter and a high-pass filter. The vortex signal was divided into three spectrum in low velocity, including $\;f_{min}\sim 2f_{min},2f_{min}\sim 4f_{min},4f_{min}\sim 8f_{min}$ and one spectrum in high velocity, including $\;8f_{min}\sim f_{max}$.$\;f_{max}\;and\;f_{min}$ are the range limit of vortex frequency under a certain diameter and medium as shown in Table 1 [26]. Taking 50 mm diameter liquid flow as an example, $\;f_{max}$= 140 Hz and $\;f_{min\;}$= 2 Hz, the frequency bands can then be divided into 2–4 Hz, 4–8 Hz, 8–16 Hz and 16–140 Hz. For a vortex signal of low frequency, its amplitude is proportional to the square of the frequency. Therefore, after the signal passing by a $1/f^{2}$ attenuation spectrum characteristics of low pass filter, the noise of high frequency is filtered and the amplitude of the signal is a constant in theory. At the same time, in order to filter the noise of low frequency better, the high-pass filter with different cut-off frequency was designed. For a vortex signal of high frequency, considering that the amplitude of the signal is saturated and the SNR (signal-to-noise ratio) is relatively high, a bandpass filter with a wide frequency band is adopted to filter out the noise out of the sub-band [26].

**Figure 4:**
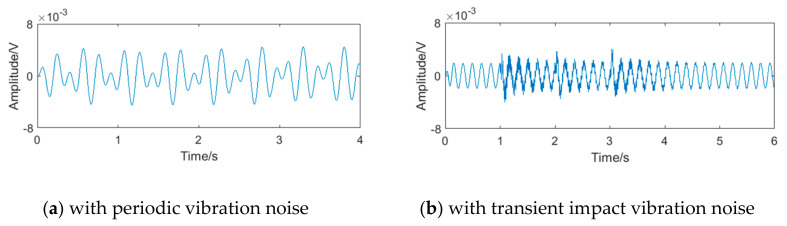
The waveform of vortex signal passing through the filter bank unit.

**Figure 5:**
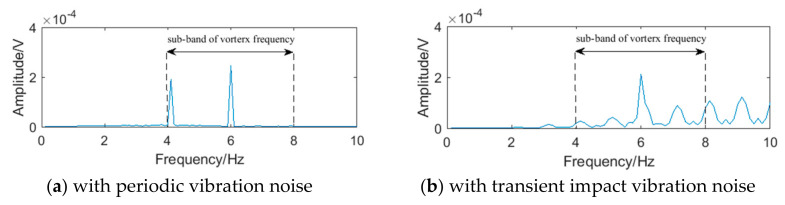
The spectrum of the vortex signal passing through the filter bank unit.

Compared with the FFT spectrum analysis method, the filter bank method has better real-time performance and lower calculation amount, which can not only improve the calculation speed but also reduce the power consumption. However, because the frequency band of the filter bank cannot be divided indefinitely, the bandwidth always exists, as shown in Figure 3. If there exists noise in the sub-band of the vortex signal, the performance of the filter bank method will be affected. Taking the 50 mm diameter liquid flow as an example, when the vortex signal with the frequency of 5.90 Hz contains periodic vibration noise or transient impact vibration noise, the signals passing through the filter bank unit of the system in literature [26] are shown in Figure 4, and its spectrums are shown in Figure 5. It can be seen that there is obvious interference in the waveform, and the interference exists in the sub-band of the vortex signal.

**Figure 6:**
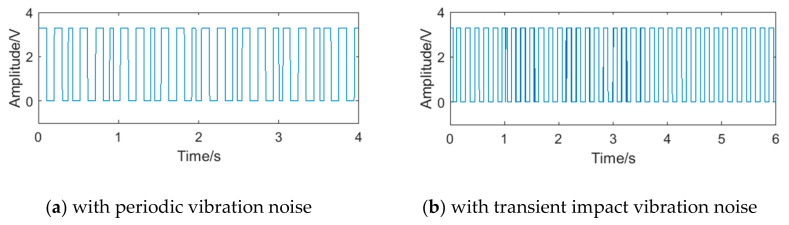
The pulse waveform of the vortex signal after pulse shaping.

**Figure 7:**
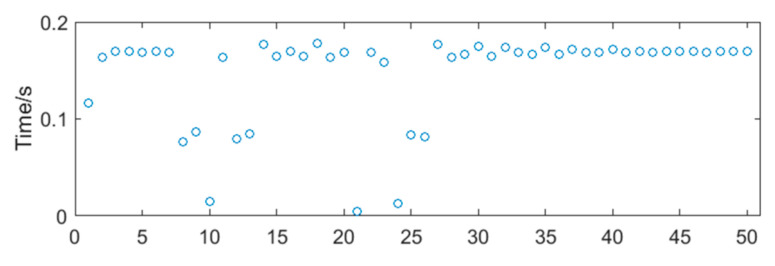
The periodic data of vortex signal with transient impact vibration noise.

The square waveforms obtained by pulse shaping of sinusoidal signals are shown in Figure 6. Because there still exist interference in the sub-band, the phenomenon of a ‘missing wave’ or ‘multiple waves’ appears, which will affect the accuracy of frequency measurement. A set of pulse period data can be obtained by counting the rising and falling edges of the square waveforms. At present, the most common method for processing periodic data in microprocessors is calculating the mean, such as the products designed by Rosement [24], Yokogawa [25] and Shanghai University [26]. Taking the vortex signal with transient impact vibration noise as an example, its periodic data is shown in Figure 7, and the relative frequency error obtained by the mean is 4.91%. The error indicated that the outliers had influence on the calculation results under the mean method, leading to the deviation of the calculation results from the overall characteristics of the data, or even no longer representative.

Therefore, it is necessary to design a signal-processing algorithm in the microprocessor to process the periodic data and further filter out the noise in the sub-band. At present, there are few frequency-correction methods in the microprocessor, which is the key to the application of the filter bank method and the design of a high-precision vortex flow meter.

To solve this problem, a frequency correction method based on the central tendency of a vortex signal is proposed in this paper. It is combined with the filter bank method to design a vortex flow meter signal processing system. The system filters out the noise out of the sub-band by the filter bank method, and then filters out the noise in the sub-band by the frequency correction method. The new method can make up for the deficiency of the FFT spectrum analysis method and filter bank method.

This paper is organized as follows. In Section 1, the essential characteristics of the vortex signal and the research status of the vortex signal-processing method are introduced. In Section 2, the generalized mode method based on the central tendency of a vortex signal is proposed, and the feasibility of the method is verified. In Section 3, the generalized mode method is combined with the filter bank method, and the system algorithm is designed. In Section 4, the simulation models of the vortex signal and noise signal are established, and the feasibility, anti-interference and real-time performance of the system are verified compared to the FFT spectrum analysis method and the filter bank method. In Section 5, the results of the anti-vibration experiment and calibration experiment are presented. In Section 6, some discussion about the experiment results are conducted. Finally, some conclusions are drawn in Section 7.

## 2. Analysis of Generalized Mode

### 2.1. The Background of Generalized Mode

**Figure 8:**
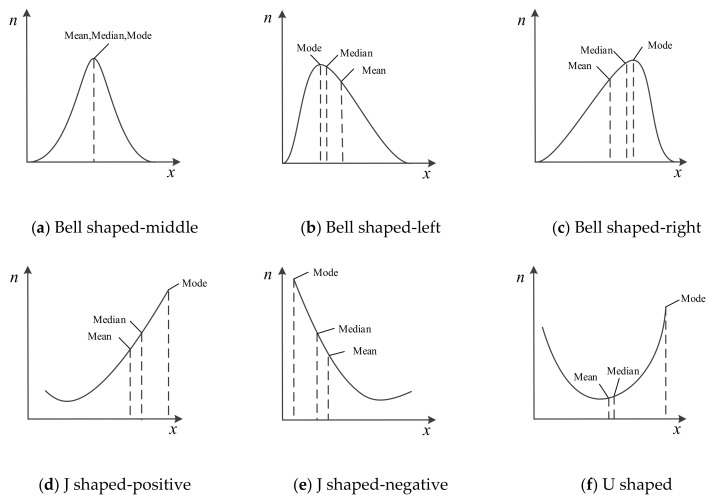
The performance of the mean, median and mode in different data distributions.

Given that the vortex signal has a good central tendency in the frequency domain, noise can be distinguished from the vortex signal and filtered by making full use of this characteristic. At present, the common methods to analyze the central tendency of data include calculating the mean, median, or mode of the collected data [27]. Their performance is different when analyzing different data distributions, as shown in Figure 8. Combined with the figure, the following conclusions can be drawn:

1. The mean represents the mean level of data, which can be calculated without data sorting. Compared with median and mode, it has good real-time performance. However, the mean is sensitive to each data and vulnerable to the influence of outliers [28,29]. In the case of skewed distribution, it will deviate from the overall characteristics of the data, or even become less representative.
2. The median represents the level of the middle position of the data, which only considers the centre position of the data set, but does not care about the difference between value of other samples and the median. Therefore, the median is robust. In the skewed distribution, it is always between the mean and the mode. Compared with the mean, outliers have less influence on it, but its selection ignores the statistical characteristics of most data.
3. The mode is the value of the data with the most frequent occurrence. It is the most direct statistic to reflect the central tendency of the data. Compared with mean and median, mode is not easily affected by outliers, while most of the statistical characteristics of data are retained. Mode adapts to the widest data distributions, which has a significant advantage in analyzing the central tendency of data.

In the process of vortex signal processing, due to the existence of noise, the periodic data of the vortex signal is not a specific value but fluctuates within a certain range, and even some outliers with large deviation may appear. At this point, the mean will affect measurement accuracy of the vortex flow meter, and the median will lose most of the periodic data conforming to the characteristics of the vortex signal. The mode may not be able to obtain meaningful values. In order to retain the advantage of mode and make it computable in actual working conditions, the definition of mode needs to be extended to get the concept of generalized mode.

The simple definition of generalized mode is as follows: compared with mode, the generalized mode is not the same number with the highest frequency. The nearest group of numbers is thought as the same number, and the mean of these numbers is called generalized mode, which is appropriate for actual working conditions.

**Figure 9:**
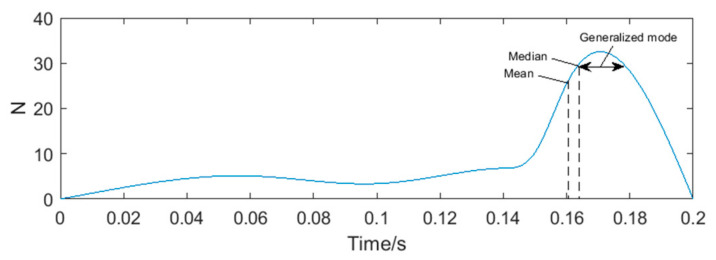
The distribution diagram of periodic data of vortex signal.

Taking the periodic data of a vortex signal with transient impact vibration noise as an example (show in Figure 7), first, the frequency of data falling in each periodic interval is counted by a histogram. The midpoint positions of the histogram are connected by a smooth curve to obtain the distribution diagram of periodic data of vortex signal, as shown in Figure 9. It approximately matches the Bell shape on the right. The mean, median, and mode of the periodic data are calculated as shown in Table 2.

### 2.2. The Definition of Generalized Mode

The generalized mode derives from the mode. In mathematical statistics, the definition of mode is that if in the data sample set $X=\left\{x_{1},x_{2},\cdots ,x_{N}\right\}$, there exists a subset $Y=\left\{y_{1},y_{2},\cdots ,y_{n}\right\}$ whose sample length is greater than one and satisfies the range $\;Rg\left(Y\right)=y_{max}-y_{min}=0$, that is $y_{1}=y_{2}=,\cdots ,=y_{n}=M$, then M is a mode of data sample set  X [30]. According to the definition of mode, it can be known that only when the public value appears in the data sample set for a sufficient number of times can the mode represent the central tendency of the data sample set well. However, in the actual measurement process, the vortex signal is vulnerable to various noises so that the measured frequency of the vortex signal is usually not a specific value that appears repeatedly but fluctuates around the accurate value [31]. Therefore, in order to retain the advantages of the mode and make it more suitable for the actual measurement of vortex signal, the concept of generalized mode is proposed in this paper.

The detailed definition of generalized mode and its related theorems, proofs, and calculation methods are given as follows.

**Definition**. *For a sample set of data that has central tendency*$X=\left\{x_{1},x_{2},\cdots x_{N}\right\}$, *among all the subset size of*n(1<n<N), *there must be a subset*$Y=\left\{y_{1},y_{2},\cdots ,y_{n}\right\}$*having the smallest*$Range\left(Y\right)=y_{max}-y_{min}$. *The subset*$Y=\left\{y_{1},y_{2},\cdots ,y_{n}\right\}$*is called the center tendency set*$Ct_{N}^{n}\;X$*and the mean value of the subset*Y*is called the generalized mode, written as*$Mg_{N}^{n}\;X$.

(3) $$Ct_{N}^{n}\;X=Y=\left\{y_{1},y_{2},\cdots ,y_{n}\right\}$$

(4) $$Mg_{N}^{n}\;X=Average\left(Y\right)=\frac{1}{n}\sum_{i=1}^{n}y_{i}$$

where $y_{max}=max\;\left(y_{1},y_{2},\cdots y_{n}\right)$, $y_{min}=min\;\left(y_{1},y_{2},\cdots y_{n}\right)$.

**Theorem** **1.** *For*$X=\left\{x_{1},x_{2},\cdots x_{N}\right\}$, *we sort it in ascending order as*$X=\left\{x_{\left(1\right)},x_{\left(2\right)},\cdots ,x_{\left(N\right)}\right\}$, *where*$x_{\left(1\right)}\leq x_{\left(2\right)}\leq \cdots \leq x_{\left(N\right)}$*. If*$x_{\left(2\right)}-x_{\left(1\right)}\geq x_{\left(N\right)}-x_{\left(N-1\right)}$*, among all the subset size of*N−1, *the subset*$Y=\left\{x_{\left(2\right)},x_{\left(3\right)},\cdots ,x_{\left(N\right)}\right\}$*with the*$x_{\left(1\right)}$*removed has the smallest*$Range\left(Y\right)=y_{max}-y_{min}$. *The subset*Y*is called the center tendency set, and the mean value of*Y*is the generalized mode of*X.

(5) $$Ct_{N}^{N-1}\;X=Y=\left\{x_{\left(2\right)},x_{\left(3\right)},\cdots ,x_{\left(N\right)}\right\}$$

(6) $$Mg_{N}^{N-1}\;X=Average\left(Y\right)=\frac{1}{N-1}\sum_{i=2}^{N}x_{\left(i\right)}$$

**Proof** **1.** The subset $Y_{1}=\left\{x_{\left(1\right)},x_{\left(2\right)},\cdots ,x_{\left(N-1\right)}\right\}$ with $x_{\left(N\right)}$ removed has its $Range\left(Y_{1}\right)=x_{\left(N-1\right)}-x_{\left(1\right)}$, and the subset $Y_{2}=\left\{x_{\left(2\right)},x_{\left(3\right)},\cdots ,x_{\left(N\right)}\right\}$ with $x_{\left(1\right)}$ removed has its $Range\left(Y_{2}\right)=x_{\left(N\right)}-x_{\left(2\right)}$. Suppose $x_{\left(2\right)}-x_{\left(1\right)}\geq x_{\left(N\right)}-x_{\left(N-1\right)}$, then $x_{\left(N\right)}-x_{\left(2\right)}\leq x_{\left(N-1\right)}-x_{\left(1\right)}$, that means $Range\left(Y_{2}\right)\leq Range\left(Y_{1}\right)$. The subset $Y_{2}=\left\{x_{\left(2\right)},x_{\left(3\right)},\cdots ,x_{\left(N\right)}\right\}$ with $x_{\left(1\right)}$ removed is the center tendency set of X. The mean value of the subset $Average\left(Y_{2}\right)$ is called the generalized mode $Mg_{N}^{N-1}\;X$ of X. □

**Theorem** **2.** *If*$x_{\left(2\right)}-x_{\left(1\right)}\leq x_{\left(N\right)}-x_{\left(N-1\right)}$*, among all the subset size of*N−1, *the subset*$Y=\left\{x_{\left(1\right)},x_{\left(2\right)},\cdots ,x_{\left(N-1\right)}\right\}$*with the*$x_{\left(N\right)}$*removed has the smallest*$Range\left(Y\right)=y_{max}-y_{min}$. *The subset*Y*is called the center tendency set, and the mean value of*Y*is the generalized mode of*X.

(7) $$Ct_{N}^{N-1}X=Y=\left\{x_{\left(1\right)},x_{\left(2\right)},\cdots ,x_{\left(N-1\right)}\right\}$$

(8) $$Mg_{N}^{N-1}\;X=Average\left(Y\right)=\frac{1}{N-1}\sum_{i=1}^{N-1}x_{\left(i\right)}$$

**Proof** **2.** The subset $Y_{1}=\left\{x_{\left(1\right)},x_{\left(2\right)},\cdots ,x_{\left(N-1\right)}\right\}$ with $x_{\left(N\right)}$ removed has its $Range\left(Y_{1}\right)=x_{\left(N-1\right)}-x_{\left(1\right)}$, and the subset $Y_{2}=\left\{x_{\left(2\right)},x_{\left(3\right)},\cdots ,x_{\left(N\right)}\right\}$ with $x_{\left(1\right)}$ removed has its $Range\left(Y_{2}\right)=x_{\left(N\right)}-x_{\left(2\right)}$. Suppose $x_{\left(2\right)}-x_{\left(1\right)}\leq x_{\left(N\right)}-x_{\left(N-1\right)}$, then $x_{\left(N-1\right)}-x_{\left(1\right)}\leq x_{\left(N\right)}-x_{\left(2\right)}$, that means $Range\left(Y_{1}\right)\leq Range\left(Y_{2}\right)$. The subset $Y_{1}=\left\{x_{\left(1\right)},x_{\left(2\right)},\cdots ,x_{\left(N-1\right)}\right\}$ with $x_{\left(N\right)}$ removed is the center tendency set of X. The mean value of the subset $Average\left(Y_{1}\right)$ is called the generalized mode $Mg_{N}^{N-1}\;X$ of X. □

The proposed strategy of generalized mode consists of the following steps:

1. Sort the data set size $N>1\;\left(N^{\prime }=N\right)$ in ascending order: $x_{\left(1\right)},x_{\left(2\right)},\cdots ,x_{\left(N-1\right)},x_{\left(N\right)}$;
2. Calculate $x_{\left(2\right)}-x_{\left(1\right)}$ and $x_{\left(N\right)}-x_{\left(N-1\right)}$;
3. If $x_{\left(2\right)}-x_{\left(1\right)}\geq x_{\left(N\right)}-x_{\left(N-1\right)}$, $x_{\left(1\right)}\;$will be discarded; otherwise, $x_{\left(n\right)}$ will be discarded. Whatever, the length of the data set N=N−1 and the data set $x_{\left(1\right)},x_{\left(2\right)},\;\cdots ,x_{\left(N-1\right)},x_{\left(N\right)}$ is updated;
4. Repeat the above steps until N=n, then the generalized mode is calculated as Equation (9).
  (9) $$Mg_{N^{\prime }}^{n}\;X=\frac{1}{n}\sum_{i=1}^{n}x_{i}$$

### 2.3. The Design of Generalized Mode Method

**Figure 10:**
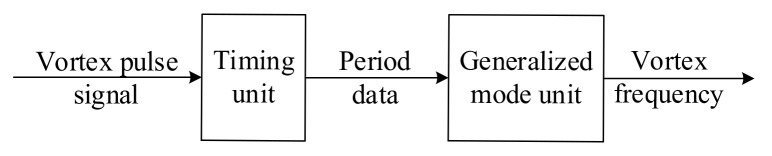
The realization process of the generalized mode method.

According to the above definition of the generalized mode, the realization of the generalized mode method is shown in Figure 10. After obtaining the vortex pulse signal, a timer is used to count the rise or fall edge of the pulses, and then the pulse period data can be obtained. After the processing of the generalized mode unit, the mean of the pulse period data is calculated, and the reciprocal of it is the frequency of the vortex signal.

**Figure 11:**
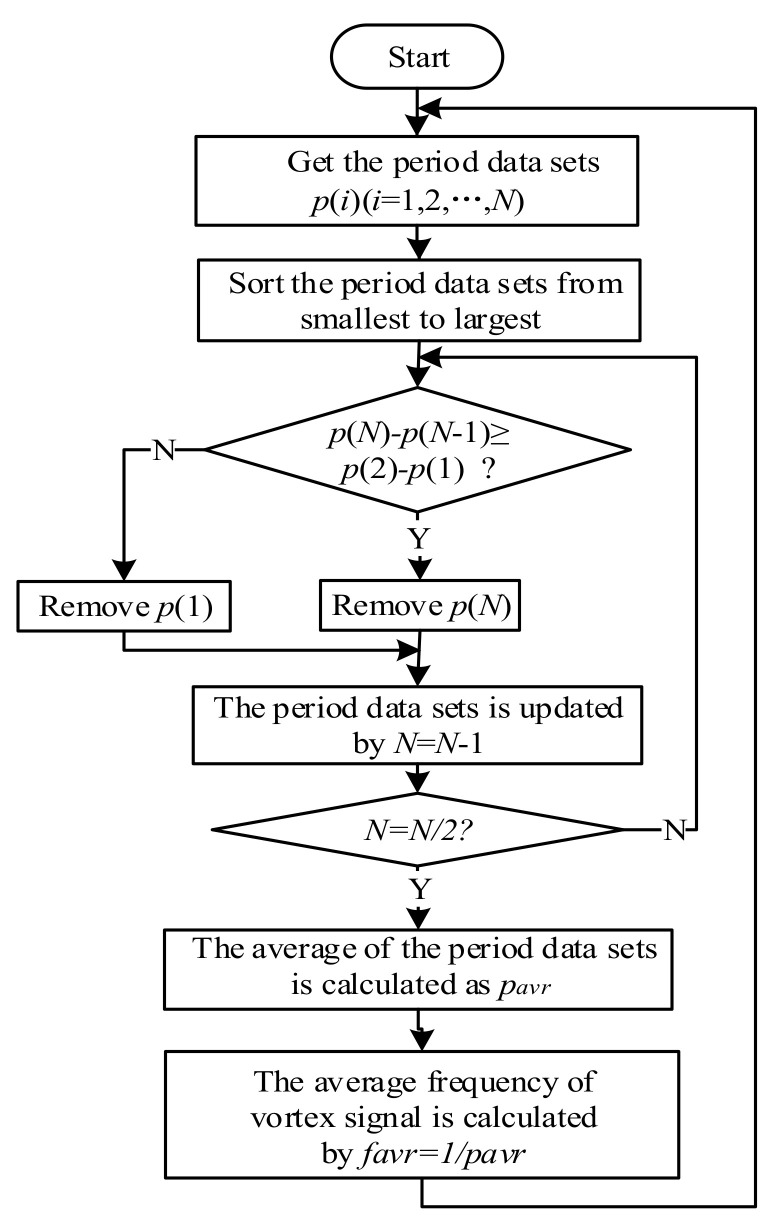
The flow chart of the generalized mode method.

Figure 11 is a flow chart of calculating the frequency of vortex signal using the generalized mode method. Set the period data sets as $\;p\left(i\right)\left(i=1,\cdots ,N\right)$. According to the generalized mode method, the period data sets are sorted from smallest to largest. $\;p\left(1\right)$ or $p\left(N\right)$ is removed by comparing $\;p\left(2\right)-p\left(1\right)$ and $p\left(N\right)-p\left(N-1\right)$, then the data sets are updated by making  N=N−1 until N reduced to n (usually take n=N/2). The mean of the n period data is $p_{avr}$, and its reciprocal is the calculated frequency of the vortex signal $f_{avr}$.

**Figure 12:**
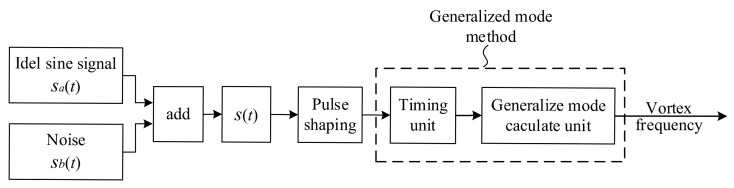
The flow chart of filtering noise in the sub-band.

To verify the feasibility of the generalized mode method to filter noise in the sub-band, a simulation experiment is designed in the MATLAB platform. The flow chart is shown in Figure 12, the ideal sine signal $S_{a}\left(t\right)$ and noise $S_{b}\left(t\right)$ are added to $\;S\left(t\right)$ as the signal to be processed. After pulse shaping, it can be transformed into the pulse signal. Then, according to the software processing of the generalized mode method, the frequency of the vortex signal is calculated.

**Figure 13:**
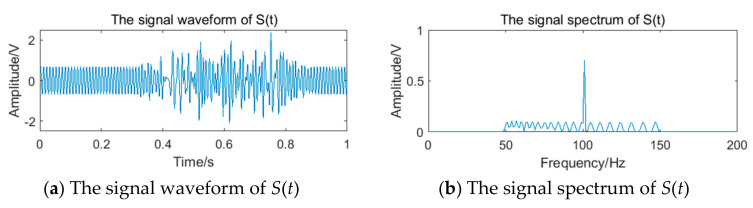
The signal waveform and spectrum of $S\left(t\right)$.

Since the time-domain synthesis algorithm of the impulse response spectrum can synthesize the corresponding time-domain waveform from a specific frequency range, it is suitable for building noise in the sub-band [32]. The $S_{a}\left(t\right)$ was taken as sinusoidal signal with a frequency of 100 Hz and an amplitude of 0.7 V, and the simulation time was 0–2 s. Suppose the range of the sub-band is 50–150 Hz, and the impulse spectrum time domain synthetic signal within this frequency range is taken as noise $S_{b}\left(t\right)$, which is superimposed between 0.3–0.9 s of the $S_{a}\left(t\right)$ as the signal $S\left(t\right)$. The signal waveform and spectrum are shown in Figure 13.

**Figure 14:**
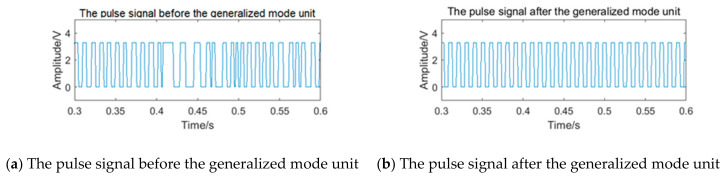
The pulse signal before and after the generalized mode unit.

After pulse shaping to the processing signal $S\left(t\right)$, the pulse signal is shown in Figure 14a. There is an obvious phenomenon of the ‘missing wave’ and ‘multi-wave’. If the frequency is calculated by averaging the pulse period data directly, error will appear. After processing the period data according to the process of the generalized mode method shown in Figure 11, the final frequency is 100.00 Hz. The pulse signal filtered by the generalized mode method is shown in Figure 14b, and the phenomenon of the ‘missing wave’ and ‘multi-wave’ is significantly improved. Therefore, the generalized mode method has a good filtering effect on the noise in the sub-band and is suitable to be used as the auxiliary filtering method of the filter bank method, to improve the measurement accuracy of vortex frequency.

## 3. System Algorithm Design

**Figure 15:**
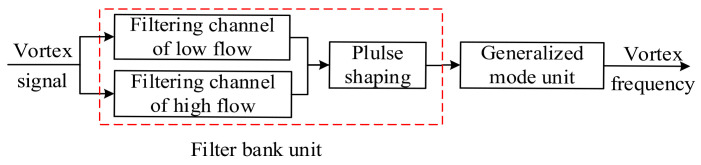
The block diagram of the overall system algorithm design.

The generalized mode method proposed in Section 2 is combined with the filter bank method to design the vortex signal-processing system. The block diagram of the overall system algorithm design is shown in Figure 15.

Firstly, the filter bank unit is composed of low-pass filters and high-pass filters in series. According to the different characteristics of a vortex signal under low and high velocity, it can be divided into the low-velocity filter channel and high-velocity filter channel as follows:

The SNR of the vortex signal with low frequency is relatively low, which means it is easily affected by noise. Therefore, it is subdivided into three filtering channels. Because the amplitude of the vortex signal with low frequency is not saturated and its amplitude is proportional to the square of the frequency, a second-order low-pass filter with $1/f^{2}$ amplitude-frequency characteristic is selected. When the vortex signal is greater than the cut-off frequency of the low-pass filter, the amplitude of the signal passing through the low-pass filter is a constant, and the high-frequency noise will be filtered out. So the cut-off frequency of the low-pass filter is set as $f_{L}=f_{min}/2$. In order to enhance the effect of filtering low-frequency noise, the cut-off frequency of the high-pass filter is set in sections. To ensure that vortex signals are not attenuated and the low-frequency noise is better filtered, the cut-off frequency of high-pass filter should be less than and as close to vortex frequency as possible. Therefore, the cut-off frequency of the high-pass filter is set as $f_{H}=f_{min},\;2\;f_{min},\;4\;f_{min}$ respectively. And the vortex signal with low frequency is divided into three frequency sub-bands as $f_{min}\sim 2\;f_{min}$, $2\;f_{min}\sim \;4\;f_{min}$ and $4\;f_{min}\sim \;8\;f_{min}$.

The SNR of a vortex signal with high frequency is relatively high, and the amplitude of the vortex signal is usually saturated. Therefore, a bandpass filter with a wide frequency band can be used to filter out the noise out of the vortex frequency band. In order to ensure the normal output of the signal and suppress high-frequency noise, the cut-off frequency of the low-pass filter is set as $f_{L}=f_{max}/2$ and the cut-off frequency of the high-pass filter is set as $f_{H}=8\;f_{min}$. And the vortex signal with high-frequency is divided into one frequency sub-band as $8\;f_{min}\sim \;f_{max}$. $f_{max}\;and\;f_{min}$ are the range limit of vortex frequency under a certain diameter and medium as shown in Table 1.

Then, the filtered signal will be converted into the pulse signal by pulse shaping, and the frequency calculation results of each channel are obtained. The filtering channel is selected according to the principle of frequency matching with the sub-band. But the signal passing through the filter bank may also have noise in the sub-band, which needs to be filtered by other filtering means.

In this paper, the generalized mode method is used to make up for the deficiency of the filter bank method by filtering noise in the sub-band. This unit makes full use of the good central tendency of a vortex signal in the frequency domain and abandons the outliers of period data formed by noise, to improve the measurement accuracy of vortex frequency.

## 4. Simulation Verification

### 4.1. The General Design of Simulation

**Figure 16:**
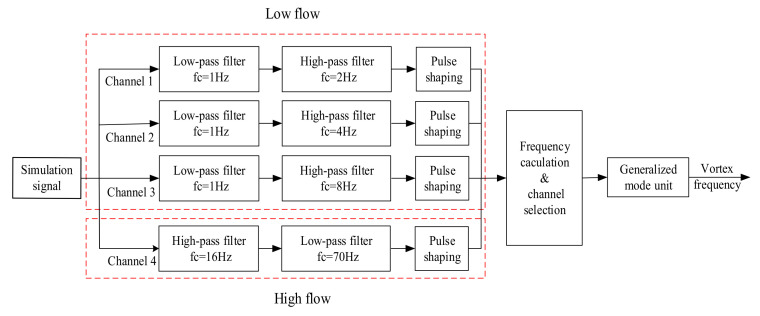
The MATLAB simulation flow chart of the system algorithm.

Taking a 50 mm diameter liquid pipe as an example, the frequency range of vortex signals is between 2 Hz and 140 Hz, according to Table 1. The vortex signal is divided into four sub-bands according to the frequency division rules of the filter bank, including 2–4 Hz, 4–8 Hz, 8–16 Hz and 16–140 Hz. Therefore, the simulation process of the system algorithm is constructed, as shown in Figure 16.

### 4.2. Feasibility Performance

**Figure 17:**
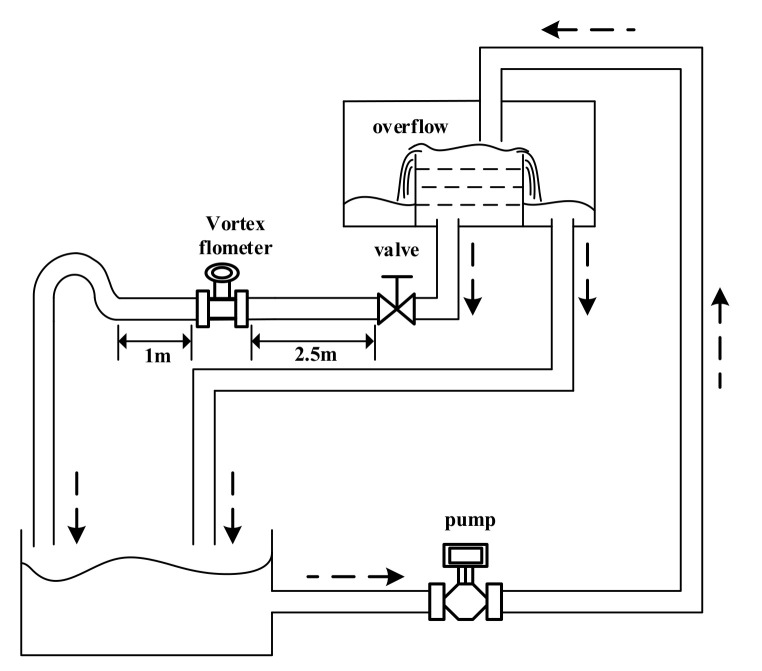
Flow field generator.

To verify the feasibility of the system, an appropriate simulation model of a vortex signal should be established first. It is known that the ideal vortex signal is a sine wave, whose frequency is proportional to the flow, and amplitude is proportional to the frequency square. The specific expression form of the relation can be measured through experiments by an experimental device, as shown in Figure 17, and the results are shown in Table 3.

The collected frequency and flow are fitted by a linear equation, and the amplitude and frequency are fitted by a quadratic equation as follows:

(10) $$Q_{V}=0.3828f_{v}$$

(11) $$A=2.5494\times 10^{-4}f_{v}{}^{2}$$

To sum up, the established simulation vortex signal needs to meet the following basic requirements:

1. It has the form of a sine wave, which satisfies the proportional relationship between frequency and flow as shown in Equation (10), including different frequency points in each channel;
2. The amplitude shown in Equation (11) is proportional to the square of the frequency.

As for the modelling of the vortex signal, the University of Oxford in the United Kingdom established a classical vortex signal model based on the analysis of signal frequency-domain power spectral density (PSD) characteristics [8]. A team in Hefei University of Technology researched the signal model based on the Oxford University study, and meanwhile considered the waveform characteristics in the time domain and amplitude attenuation phenomena. The literature [33] collected a large number of vortex signal, and established the modulation signal model used to describe the vortex signal. Its mathematical expression is as follows:

(12) $$y\left(t\right)=\left(A_{0}+K_{a}\delta_{a}\left(t\right)\right)\sin\left(2\pi f_{0}t+K_{f}\int \delta_{f}\left(\tau \right)d\tau +\varphi_{0}\right)+n\left(t\right)$$

where, $f_{0}$ and $A_{0}$ are the ideal frequency and amplitude of vortex signal respectively;$\;K_{f}$ and $K_{a}$ are the FM sensitivity and AM sensitivity respectively, $\;K_{a}\approx 1,K_{f}\approx f_{0}/f_{s}$, $f_{s}$ is the sampling frequency of the vortex signal; $\;\delta_{a}\left(t\right)$ and $\delta_{f}\left(t\right)$ are instantaneous deviations of the frequency and amplitude of the vortex signal, which are approximated by the Gaussian white noise with the expected mean value of $f_{0}$ and $A_{0}$ and the standard deviation of $\sigma =c/\sqrt{2}$; $\;\varphi_{0}$ has little influence on the extraction of vortex signal frequency, so $\varphi_{0}=0$; $\;n\left(t\right)$ refers to other noise interference, including random noise, 50 Hz power frequency noise and low-frequency oscillation noise of the 1/5–1/10 vortex frequency, which can be simulated by a random signal and sinusoidal signal, respectively.

Taking the 41 Hz vortex signal in a 50 mm diameter liquid pipe as an example, the amplitude sequence and frequency sequence of the collected vortex signal are extracted, and the probability density analysis is performed on them by the kernel density estimation method [34]. Then, Gaussian fitting is performed on the PDF (Probability Density Function) of amplitude and frequency sequence, respectively. The relevant fitting parameters are shown in Table 4.

**Figure 18:**
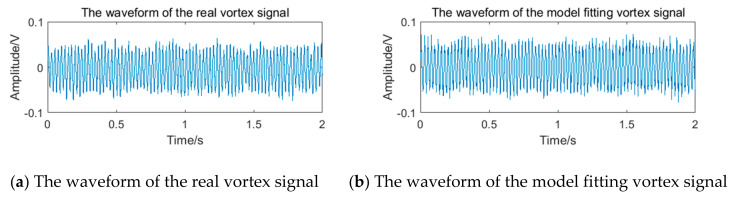
The waveform contrast diagram of the real vortex signal and the model-fitting vortex signal.

By substituting the above parameters into the model formula shown in Equation (12), the simulated vortex signal can be obtained and compared with the real vortex signal, as shown in Figure 18. The mean amplitude spectrum and PFD estimation are used to evaluate the effect of model fitting [33]. The calculated error of mean amplitude spectrum e=0.85%, and the PDF correlation coefficient $\rho_{xy}=0.985$ is very close to 1. Therefore, it can be judged that the model fits the real vortex signal well.

Literature [33] showed that $\delta_{a}\left(t\right)$ and $\delta_{f}\left(t\right)$ measured on the same vortex flow meter at different frequencies approximately satisfy the relation of the quadratic function. Therefore, $\delta_{a}\left(t\right)$ and $\delta_{f}\left(t\right)$ of different frequency vortex signals are obtained to fitting the relation as follows:

(13) $$\sigma_{a}=3.315\times 10^{-6}f_{0}{}^{2}+1.608\times 10^{-5}f_{0}+5.771\times 10^{-5}$$

(14) $$\sigma_{f}=1.255\times 10^{-3}f_{0}{}^{2}-1.754\times 10^{-2}f_{0}+0.8136$$

To sum up, taking the signal in 50mm diameter liquid pipe as example, 4 frequency points are selected in 4 channels to generate simulation vortex signals. The chosen frequency points are substituted into Equations (11), (13) and (14) in turn to get the model parameters, as shown in Table 5.

**Figure 19:**
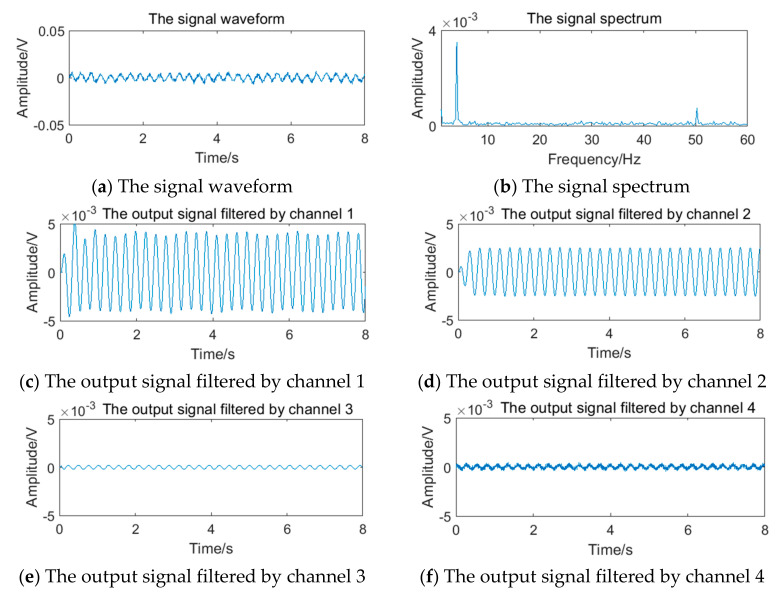
Vortex signal and the results of the filter bank of (3.77 Hz).

**Figure 20:**
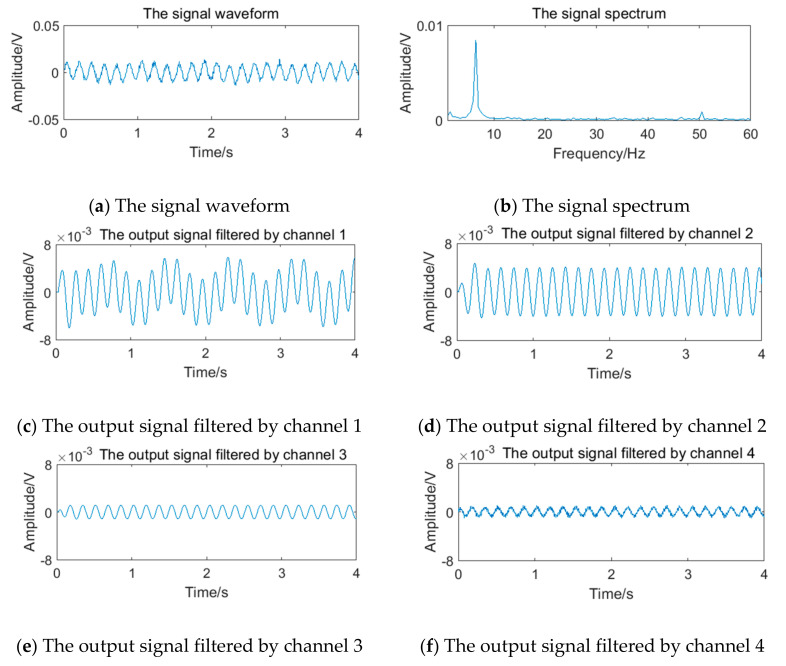
Vortex signal and the results of the filter bank (5.90 Hz).

**Figure 21:**
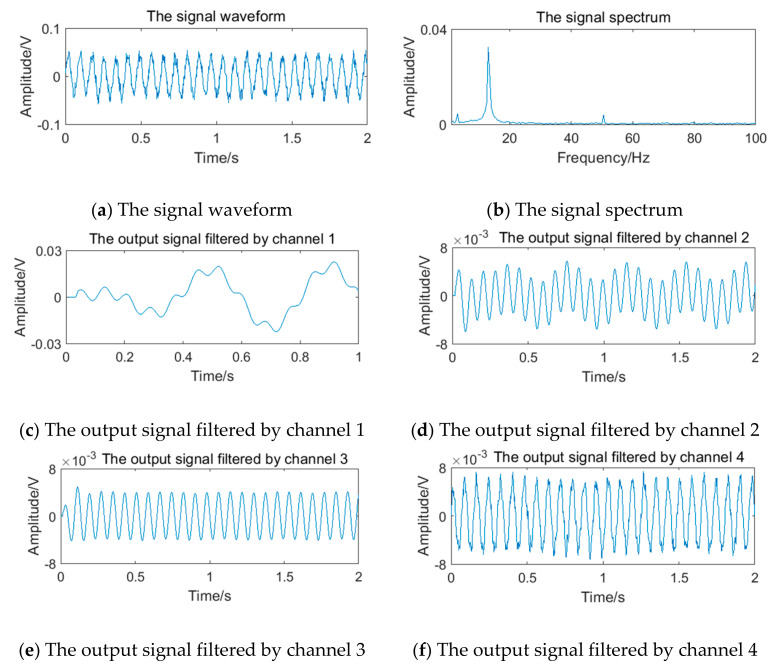
Vortex signal and the results of the filter bank (12.69 Hz).

**Figure 22:**
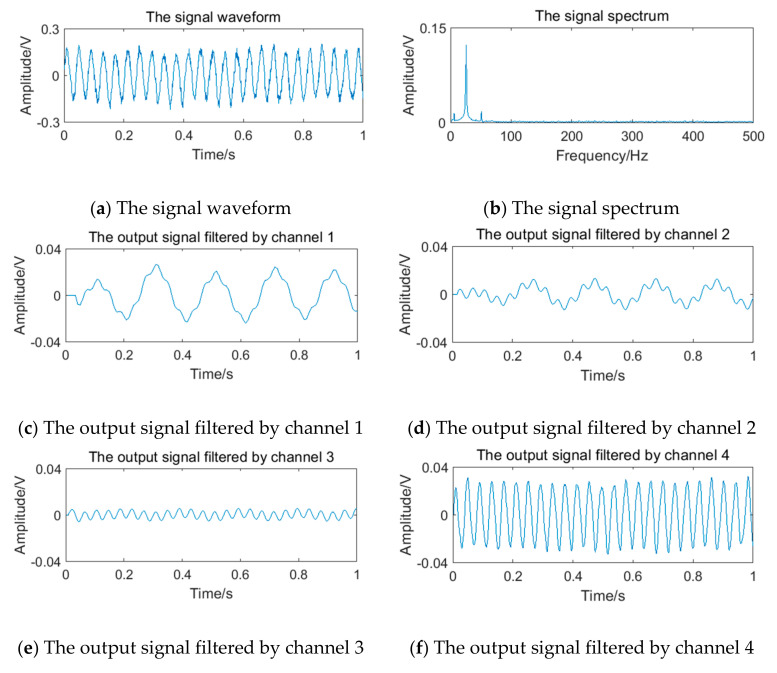
Vortex signal and the results of the filter bank (24.64 Hz).

The relevant parameters in Table 5 are substituted into Equation (12) to obtain the simulation vortex signals. According to the simulation framework of the system algorithm shown in Figure 16, vortex signals of 4 frequency points should be filtered by four channels of filter bank, respectively. The waveform and spectrum of simulation vortex signals and the output waveform of signals filtered by the filter bank are shown in Figure 19, Figure 20, Figure 21 and Figure 22.

After pulse shaping, the results of frequency calculation and sub-band selection of four filter bank channels are obtained, as shown in Table 6.

Pulse signal of the selected channel is input into the generalized mode unit for further filtering, and the final frequency calculation result and relative error are obtained. Meanwhile, the bilateral correction method, the ‘double-window’ method, and the filter bank method proposed in [15,20], and [4], respectively, are compared here, and the results are shown in Table 7.

### 4.3. Anti-Interference Performance

Deterministic interference such as random noise, power frequency noise, and low-frequency oscillations noise have been considered in the vortex signal model in 4.2. For uncertain interference, such as periodic vibration noise and transient impact vibration noise, reference [33] and reference [35] established the simulation models of noise, and their mathematical expressions are shown in Equations (15) to (17). To sum up, the established simulation noise needs to meet the following basic requirements:

1. Including all kinds of possible interference in vortex signal, to truly restore the field measurement environment and verify the anti-interference performance of the system under complex working conditions.
2. The periodic vibration noise conforms to the model shown in Equation (15), and the noise frequency and intensity can be changed by adjusting the parameters $f_{n}$ and $A_{n}$ respectively.
  (15) $$y_{n}\left(t\right)=A_{n}\left(t\right)\sin\left(2\pi f_{n}t+\varphi_{n}\right)+n’\left(t\right)$$
3. The transient impact vibration noise conforms to the model shown in Equations (16) and (17), and the times of noise can be changed by signal superposition.
  (16) $$x\left(t\right)=\sum_{i=1}^{n}x_{i}\left(t\right)+n_{e}\left(t\right)$$
  (17) $$x_{i}\left(t\right)=a_{i}+b_{i}e^{-\xi_{i}t}\sin\left(2\pi f_{i}t+\varphi_{i}\right)$$

**Figure 23:**
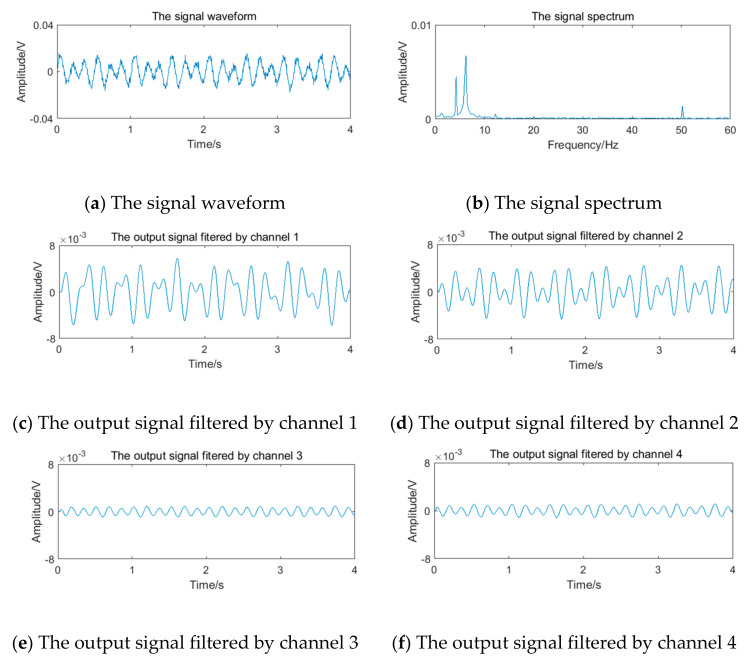
Vortex signal with periodic vibration noise and the results of filter bank (5.90 Hz).

The vortex signal of 5.90 Hz generated in 4.2 is superimposed with the periodic vibration noise of different frequency and intensity. Taking the vortex signal with weak periodic vibration noise of 4 Hz as an example, the stimulation signal and the results of the filter bank are shown in Figure 23. It can be seen that the filter bank cannot completely overcome the influence of periodic vibration noise.

**Figure 24:**
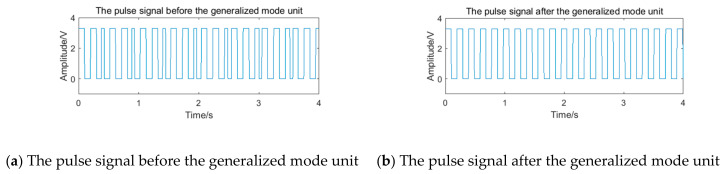
The pulse signal with periodic vibration noise before and after the generalized mode unit.

After pulse shaping, the selected signal of channel 2 is further input into the generalized mode unit. The pulse signal before and after the generalized mode unit is shown in Figure 24. Because the noise in the sub-band still exists, the pulse signal appears to be the obvious ‘missing wave’ and ‘multi-wave’ phenomenon, which will cause errors in frequency calculating. After passing through the generalized mode unit, the phenomenon of ‘missing wave’ and ‘multi-wave’ is improved.

The relative errors of vortex signal frequency under different periodic vibration noise conditions and signal processing methods are obtained, as shown in Table 8. The frequency sub-band of vortex signal is 4–8 Hz, so that the noises of 40 Hz are out of the sub-band, and the noises of 4 Hz are in the sub-band.

**Figure 25:**
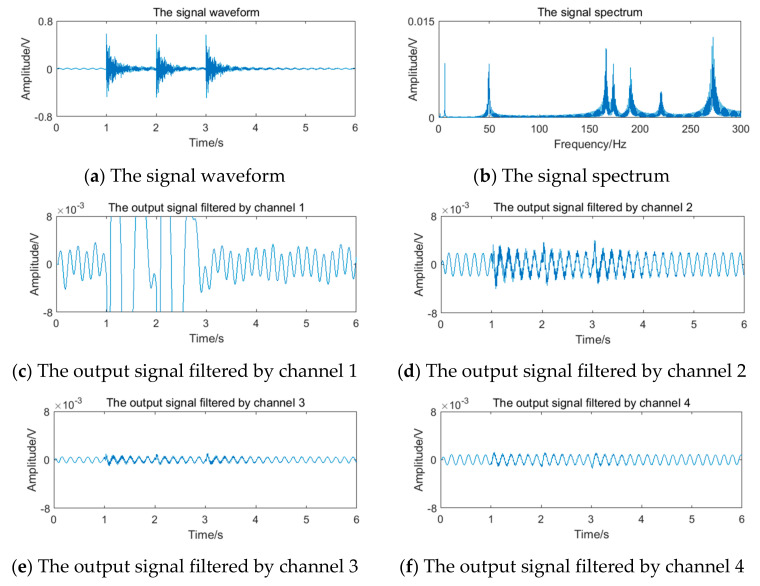
Vortex signal with transient impact vibration noise and the results of the filter bank (5.90 Hz).

Similarly, the vortex signal of 5.90 Hz generated in 4.2 is superimposed with the transient impact vibration noise. Taking vortex signal with strong transient impact vibration noise of 3 times as an example, the simulation signal and the results of the filter bank are shown in Figure 25. It can be seen that the energy of the transient impact vibration noise exceeds the vortex signal, and its spectrum is complicated, including the frequency in different sub-bands. Therefore, the filter bank cannot completely overcome the influence of transient impact vibration noise.

**Figure 26:**
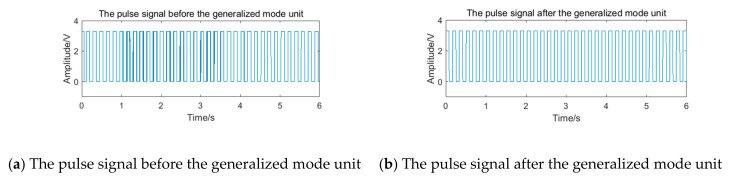
The pulse signal with transient impact vibration noise before and after the generalized mode unit.

After pulse shaping, the selected signal of channel 2 is further input into the generalized mode unit. The pulse signal before and after the generalized mode unit is shown in Figure 26**.** Because the noise in the sub-band still exists, the pulse signal also appears as the obvious ‘missing wave’ and ‘multi-wave’ phenomenon, which will cause errors in frequency calculating. After passing through the generalized mode unit, the phenomenon of ‘missing wave’ and ‘multi-wave’ is improved.

The relative errors of vortex signal frequency under different transient impact vibration noise conditions and signal processing methods are obtained, as shown in Table 9.

### 4.4. Real-Time Performance

According to the previous analysis, the FFT spectrum analysis method is sensitive to data length, which is an essential factor affecting the real-time performance of a vortex flow meter. Four kinds of simulation signal are built to compare the generalized mode method with the two FFT spectrum analysis method [15,20]. The measurement results are shown in Table 10.

## 5. Experimental Verification

### 5.1. Anti-Vibration Experiment

**Figure 27:**
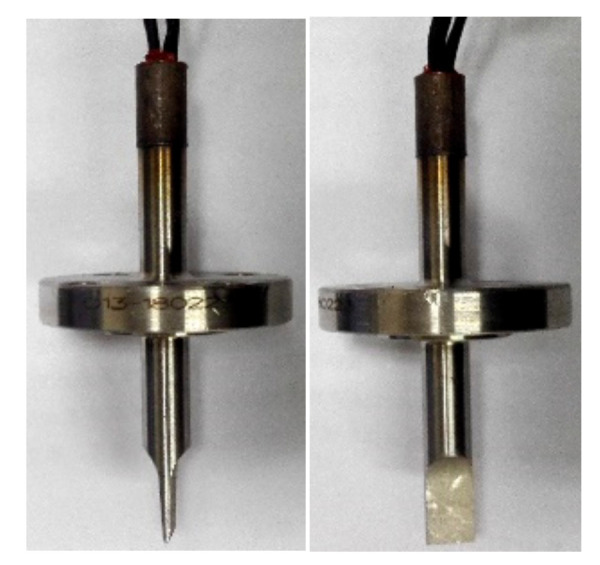
The piezoelectric sensor with a separated cantilever structure.

A piezoelectric sensor plays an important role in the measurement of a vortex signal. In this paper, a piezoelectric sensor with a separated cantilever structure, as shown in Figure 27, is used for the experiment. In the piezoelectric sensor, the piezoelectric element is encapsulated within it. When the fluid flows through the vortex generator, vortices will be formed and strike alternately on both sides of the flat body. The piezoelectric element in the piezoelectric sensor will be deformed when it is subjected to alternating pressure generated by the vortices, and the pressure signal will be converted into an electric signal. The electric signal will be processed by the system to measure flow in the pipeline.

**Figure 28:**
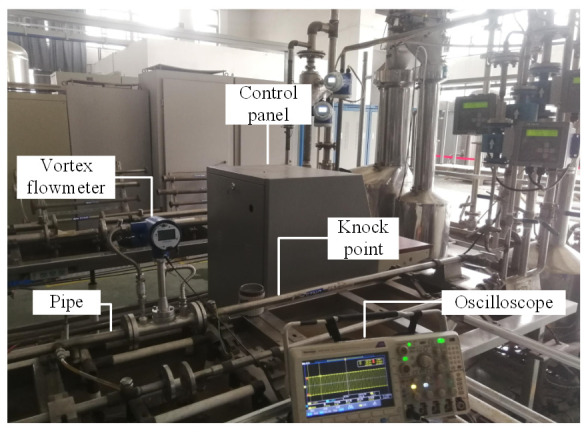
The field diagram of the anti-vibration experiment.

The algorithm proposed in this paper is implemented by using STM32F103VET6 (STMicroelectronics, Geneva, Switzerland) as the controller with a system clock of 2 MHz. We install the vortex flow meter designed in this paper in the 40 mm diameter liquid flow pipe, and then knock the pipe evenly. Figure 28 is the field diagram of the anti-vibration experiment.

**Figure 29:**
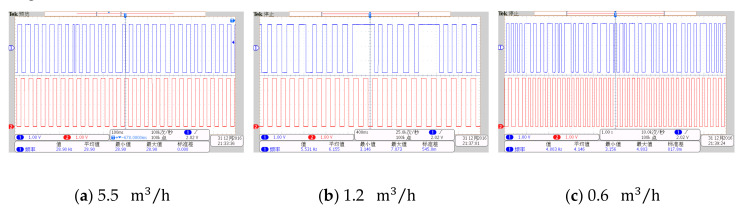
The waveform passing through the filter bank unit and the generalized mode unit.

During the interference, the pulse waveforms passing through the filter bank unit and the generalized mode unit are simultaneously collected by the oscilloscope. Figure 29 is a comparison diagram of three groups of signals at different flow rates. In each diagram, the upper part is the waveform passing through the filter bank unit, and the lower part is the waveform passing through the generalized mode unit.

It can be seen that at different flow rates, the interference generated by knocking has a great impact on the system. After passing through the filter bank unit, an obvious phenomenon of ‘missing wave’ or ‘multi-wave’ appears in the waveform. After the frequency correction of the generalized mode unit, the phenomenon of ‘missing wave’ or ‘multi-wave’ is improved, which indicates that the generalized mode method has good anti-interference performance in the actual situation.

### 5.2. Calibration Experiment

The vortex flow meter designed in this paper was calibrated under an 80 mm diameter gas pipe, and the calibration result is shown in Table 11. It can be seen that the repeatability error of the algorithm is less than 0.14%.

## 6. Results Discussion

The feasibility is shown in Table 7; the 4 algorithms proposed in [4,15,20] and this paper all perform well when there is no obvious noise, and the absolute value of the relative error of the four methods is less than 1%. Furthermore, the absolute value of the relative error of the algorithm proposed in this paper is less than 0.31%, indicating that the method combining filter bank with generalized mode can extract the frequency of vortex signals accurately.

The anti-interference is shown in Table 8 and Table 9. It can be seen from Table 8, for the periodic vibration noise in the sub-band, especially the strong periodic vibration noise, the performance of the filter bank method [4] is affected, and the relative frequency error is –3.47%. The algorithm proposed in this paper can deal with the periodic vibration noise in the sub-band better compared to the filter bank method [4]. It can be seen from Table 9 that the performance of the FFT spectrum analysis method [15,20] will be affected greatly when there is strong transient impact vibration noise because the peak point of the spectrum corresponds to noise. With the increase of the intensity and frequency of the transient impact vibration noise, the relative error caused by the filter bank method [4] also increases. While the absolute value of the relative error of the algorithm proposed in this paper keep within 1% under various noise conditions, which means the system has better anti-interference performance compared to the FFT spectrum analysis method [15,20], and the filter bank method [4].

The real-time performance is shown in Table 10. When there are enough samples, the measurement accuracy of the FFT spectrum analysis method is high. However with the decrease of samples, the measurement accuracy of the FFT spectrum analysis method is decreased. Especially in the case of a small sample size, the measurement error of the vortex signal with noise in the sub-band is relatively large. The algorithm proposed in this paper is not sensitive to the sample size, which means it has better real-time performance compared to the FFT spectrum method [15,20].

## 7. Conclusions

1. A new central tendency analysis method called the generalized mode algorithm is proposed in this paper. Compared with the traditional mean, median, and mode, the generalized mode is insensitive to outliers. It retains most of the statistical characteristics of the data, which is more suitable for practical application. In addition to being used in the vortex flow meter signal-processing system, the idea of generalized mode can also be used for reference in other signal-processing fields, such as the electromagnetic flow meter signal-processing system.
2. A new signal-processing method of vortex flow meter is designed based on the generalized mode algorithm, which avoids the conflict between frequency resolution and real-time performance existing in the FFT spectrum analysis method. Meanwhile, it solves the problem that the filter bank method needs to filter out the noise in the sub-band. Experiments show that the proposed algorithm has good measurement accuracy and anti-interference performance, especially for the interference of strong periodic vibration noise and strong transient impact vibration noise in the sub-band. Under various interference conditions, the absolute value of the relative error of vortex frequency is within 0.87%, as shown in Table 7, Table 8, Table 9 and Table 10.
3. The generalized mode method is applied in the vortex flow meter system. The results of the anti-vibration experiments carried out in the 40 mm diameter liquid flow device showed that the generalized mode method performed better than the filter bank method, as shown in Figure 28. The results of the calibration experiments in the 80 mm diameter gas flow device showed that the repeatability of the vortex flow meter designed in this paper is less than 0.14%, as shown in Table 11, which indicates it has good practical application value.

## Figures and Tables

**Table 1:** The frequency measurement range under different diameter and medium.

Diameter (mm)	Frequency Range of Liquid (Hz)	Frequency Range of Gas (Hz)
25	8.75–270	45.00–2650
32	5.00–220	32.50–2200
40	2.50–180	22.50–1650
50	2.00–140	20.00–1400
65	1.75–110	15.00–1100
80	1.50–90	10.00–850
100	1.25–75	7.50–720
125	1.13–58	5.75–580
150	0.95–50	4.50–460
200	0.80–40	3.25–380
250	0.63–31	2.75–260

**Table 2:** The central tendency indicators of the periodic data.

Index	Period (s)	Frequency (Hz)	The Relative Error (%)
Mean	0.1616	6.19	4.91
Median	0.1672	5.98	1.36
Mode	\	\	\
Generalized mode	0.1697	5.89	–0.1

**Table 3:** The flow, frequency, and amplitude of a vortex signal in a 50 mm diameter pipe.

Flow (L/s)	Frequency (Hz)	Amplitude (mV)
2.02	5.27	7.08
2.92	7.64	14.88
8.99	23.48	140.55
11.99	31.33	250.24
17.76	46.39	548.64
2.02	5.27	7.08

**Table 4:** The Gauss fitting parameters.

PDF	*a* ^1^	*b* ^2^	*c* ^3^	Degree of Fitting	Ideal Value	σ ^4^
Amplitude	62.8	0.05249	0.00890	0.9971	0.0515 V	0.00629
Frequency	0.1767	40.83	3.117	0.9916	40.89 Hz	2.20405

*a*^1^, *b*^2^, *c*^3^ are the Gaussian fitting parameters from $y_{g}=a\times e^{-\frac{{\left(x_{g}-b\right)}^{2}}{c^{2}}}$ and  σ^4^ $=c/\sqrt{2}$.

**Table 5:** The model parameters of 4 channels.

Channel	Sub-Band	Frequency (Hz)	Amplitude (V)	$\sigma_{a}$	$\sigma_{f}$
1	2–4	3.77	0.0036	0.0002	0.7653
2	4–8	5.90	0.0089	0.0003	0.7538
3	8–16	12.69	0.0411	0.0008	0.7931
4	16–140	24.64	0.1548	0.0025	1.1434

**Table 6:** The results of frequency calculation and sub-band selection of 4 channels.

Frequency Point (Hz)	Channel 1 (2–4 Hz)	Channel 2 (4–8 Hz)	Channel 3 (8–16 Hz)	Channel 4 (16–140 Hz)	Selected Sub-Ban (Hz)
3.77	3.7500	3.7975	0.0000	0.0000	2–4
5.90	5.7391	5.8428	0.0000	0.0000	4–8
12.69	4.9485	12.2034	12.7946	12.8142	8–16
24.64	5.1173	11.8812	23.8914	24.5629	16–140

**Table 7:** The relative errors of vortex signal with different frequencies.

Frequency Point (Hz)	Relative Error (%)			
	[20]	[15]	[4]	Ours
3.77	0.40	0.27	−0.53	0.31
5.90	0.34	0.13	−0.97	−0.17
12.69	−0.32	−0.18	0.82	0.15
24.64	−0.14	−0.06	0.31	0.11

**Table 8:** The relative errors of vortex signal with different periodic vibration noise.

Noise Frequency (Hz)	Noise Intensity	Relative Error (%)			
		[20]	[15]	[4]	Ours
4	Weak	0.43	–0.34	–1.10	–0.38
	Strong	–0.61	0.79	–3.47	0.85
40	Weak	–0.19	–0.15	–0.28	0.17
	Strong	0.58	0.35	0.44	–0.25

**Table 9:** The relative errors of vortex signal with different transient impact vibration noise.

Noise Intensity	Noise Times	Relative Error (%)			
		[20]	[15]	[4]	Ours
Weak	1	0.31	0.17	–0.57	–0.18
	2	–0.37	0.35	1.06	0.21
	3	0.46	–0.30	4.91	0.29
Strong	1	>100	>100	2.02	–0.38
	2	>100	>100	3.40	0.65
	3	>100	>100	5.52	0.78

**Table 10:** The measurement results of the three algorithms.

Vortex Signal	Noise Signal	Cycle Number	Relative Error (%)		
			[20]	[15]	Ours
Low flow	Out of the sub-band	40	0.00	0.00	0.00
		20	0.00	0.00	0.00
		10	0.00	0.02	0.00
		5	0.11	−0.15	0.00
		3	−1.69	−2.04	−0.23
Low flow	In the sub-band	40	0.00	0.00	0.00
		20	0.00	0.00	0.01
		10	0.00	−0.03	−0.12
		5	0.64	0.96	0.52
		3	4.78	5.21	−0.72
High flow	Out of the sub-band	40	0.00	0.00	0.00
		20	0.00	0.00	0.00
		10	0.01	0.00	0.02
		5	−0.15	−0.19	−0.08
		3	0.25	0.37	−0.10
High flow	In the sub-band	40	0.00	0.00	0.00
		20	0.00	0.00	0.00
		10	−0.01	−0.05	0.01
		5	0.98	0.31	−0.20
		3	−3.01	−4.49	0.87

**Table 11:** The calibration results of 80 mm diameter gas flow.

Location of the Flow Detection (m^3^/h)	Testing Time (s)	Total Pulse (Pulse)	Mean Frequency (Hz)	Standard Values (m^3^/h)	K Factor ^1^ (1/L)	Mean Coefficient (1/L)	Repeatability Error (%)
63	60.000	2412	40.200	63.457	2.2806	2.2772	0.13
	59.000	2366	40.102	63.457	2.2749		
	59.000	2367	40.119	63.457	2.2761		
143	59.000	5338	90.475	143.191	2.2749	2.2763	0.09
	60.000	5438	90.633	143.194	2.2786		
	59.000	5340	90.508	143.189	2.2753		
302	60.000	11420	190.333	301.992	2.2689	2.2712	0.14
	59.000	11260	190.847	301.992	2.2750		
	60.000	11424	190.400	301.985	2.2698		
548	59.000	20375	345.339	547.679	2.2699	2.2676	0.11
	59.000	20355	345.000	547.679	2.2678		
	59.000	20331	344.593	547.676	2.2651		
695	59.000	25665	435.000	694.701	2.2542	2.2570	0.12
	60.000	25704	428.400	694.722	2.2576		
	59.000	26160	443.390	694.723	2.2593		

^1^ K factor is sometimes called ‘pulses per liter’ [10].
